# A Digital Counselor-Delivered Intervention for Substance Use Among People With HIV: Development and Usability Study

**DOI:** 10.2196/40260

**Published:** 2023-08-28

**Authors:** Heidi E Hutton, Saavitri Aggarwal, Afroza Gillani, Geetanjali Chander

**Affiliations:** 1 Department of Psychiatry & Behaviorial Science Johns Hopkins University School of Medicine Baltimore, MD United States; 2 College of Dentistry New York University New York, NY United States; 3 Division of General Internal Medicine University of Washington School of Medicine Seattle, WA United States

**Keywords:** computer-delivered intervention, HIV, substance use, virtual counselor, motivational interviewing, people with HIV, substance misuse, virtual intervention, evidence-based care, engagement, health outcome, support, behavioral intervention, medical counseling, patient care, virtual care, counseling, mHealth, telecounseling

## Abstract

**Background:**

Substance use disorders are prevalent and undertreated among people with HIV. Computer-delivered interventions (CDIs) show promise in expanding reach, delivering evidence-based care, and offering anonymity. Use in HIV clinic settings may overcome access barriers. Incorporating digital counselors may increase CDI engagement, and thereby improve health outcomes.

**Objective:**

We aim to develop and pilot a digital counselor–delivered brief intervention for people with HIV who use drugs, called “C-Raven,” which is theory grounded and uses evidence-based practices for behavior change.

**Methods:**

Intervention mapping was used to develop the CDI including a review of the behavior change research in substance use, HIV, and digital counselors. We conducted in-depth interviews applying the situated-information, motivation, and behavior skills model and culturally adapting the content for local use with people with HIV. With a user interaction designer, we created various digital counselors and CDI interfaces. Finally, a mixed methods approach using in-depth interviews and quantitative assessments was used to assess the usability, acceptability, and cultural relevance of the intervention content and the digital counselor.

**Results:**

Participants found CDI easy to use, useful, relevant, and motivating. A consistent suggestion was to provide more information about the negative impacts of drug use and the interaction of drug use with HIV. Participants also reported that they learned new information about drug use and its health effects. The CDI was delivered by a “Raven,” digital counselor, programmed to interact in a motivational interviewing style. The Raven was perceived to be nonjudgmental, understanding, and emotionally responsive. The appearance and images in the intervention were perceived as relevant and acceptable. Participants noted that they could be more truthful with a digital counselor, however, it was not unanimously endorsed as a replacement for a human counselor. The C-Raven Satisfaction Scale showed that all participants rated their satisfaction at either a 4 (n=2) or a 5 (n=8) on a 5-point Likert scale and all endorsed using the C-Raven program again.

**Conclusions:**

CDIs show promise in extending access to care and improving health outcomes but their development necessarily requires integration from multiple disciplines including behavioral medicine and computer science. We developed a cross-platform compatible CDI led by a digital counselor that interacts in a motivational interviewing style and (1) uses evidence-based behavioral change methods, (2) is culturally adapted to people with HIV who use drugs, (3) has an engaging and interactive user interface, and (4) presents personalized content based on participants’ ongoing responses to a series of menu-driven conversations. To advance the continued development of this and other CDIs, we recommend expanded testing, standardized measures to evaluate user experience, integration with clinician-delivered substance use treatment, and if effective, implementation into HIV clinical care.

## Introduction

Substance use disorders affect approximately 48% of people with HIV and have a significant negative impact on HIV disease throughout the care continuum. They worsen engagement and retention in care, negatively affect medication adherence and viral suppression, and increase morbidity and mortality [1]. Thus, optimal HIV care includes substance use treatment; however, there are several barriers to the delivery of care in clinics including inadequate provider time and training, and service costs [2]. Among clients, the barriers include anticipated stigma about substance use treatment, uncertainty about and availability of treatment options, lack of perceived need for treatment, cost, and transportation [3-5].

Computer-delivered interventions (CDIs) can offer potential solutions to these barriers.

CDI can reach large numbers of clinic patients, are perfectly replicable, offer greater anonymity, and appear cost-effective [6-10]. They also can be readily adapted to patient sociodemographic characteristics because the content is modular and menu driven [11]. Three recent systematic reviews and meta-analyses indicate that across diverse settings (emergency room, primary care, specialty addiction center, and university), CDIs have been effective in improving treatment engagement and skill acquisition and in changing behavior compared with treatment as usual [8-10]. Most studies, however, have focused on alcohol use and on younger populations, particularly college populations [10]. In the few studies comparing CDI with clinician-delivered counseling, 2 were found to be equally effective [12,13] and in a third CDI produced greater treatment satisfaction and a greater number of biologically confirmed abstinence days [14]. Notably, the CDI condition included a person-delivered, brief (10-minute) weekly monitoring sessions. Although the authors reported that these sessions adhered to guidelines for low-intensity interventions used in placebo-controlled trials, it does suggest that at least a modicum of organizational support may be necessary for improved outcomes.

Smartphone apps show some promise in reducing substance use among adults but the evidence is far from conclusive [15]. Among people with HIV, CDIs for substance use have had very limited development with the exception of HealthCall [16], which was effective in reducing number of noninjection drug use days among people with HIV and could deliver treatment on demand. The disadvantage of smartphones, as well as tablets and computers, is that they are expensive and expensive to replace [10]. Additionally, marked disparities in internet use continue to exist even as the use of these devices is increasing among low-income populations across the United States [17]. Importantly, for many people substance use is a chronic relapsing condition that requires continuing care to improve outcomes [18]. Digital interventions beyond simple text messaging that can also ensure intervention delivery and retention in care may require sustained organizational support [10,14,19]. As such, using clinic-administered CDI may increase access, engagement, and continuity of treatment for people with HIV.

Recent innovative approaches to increase CDI efficacy include the use of digital counselors (also known as embodied conversational agents or digital agents) to interface between intervention and users [20,21]. Preliminary research has shown that digital counselors for substance use interventions are not only evaluated as acceptable and helpful but also empathic and likable [22,23]. Empathy, defined as the understanding of another person’s experience, is particularly vital as it is consistently linked to improved client outcomes in person-delivered interventions [24]. For this reason, the principles of motivational interviewing (MI) are frequently incorporated into the repertoire of digital agents [25]. In alcohol interventions, an empathic and likable digital counselor versus one low in empathy was rated as more supportive and understanding, and increased intention to reduce alcohol [26] and decreased alcohol use over a 3 month assessment period [27].

Across a variety of behavior change interventions, digital counselors can improve engagement, knowledge, and enjoyment [28]. In alcohol screening, patients at US Veterans Administration medical center were more likely to disclose alcohol use to a digital agent than to a research assistant and were more satisfied with the alcohol assessment when it was delivered by the agent than by text-based assessment [29]. Digital counselor–led CDIs have been effective in reducing biologically confirmed illicit drug use among postpartum women [30] (although not consistently) [31]. Digital counselors may improve engagement and retention in substance use treatment which are so essential to optimizing clinical outcomes [32,33].

The development and application of CDIs are rapidly increasing and evolving. As the field expands, documentation of intervention development is needed to understand the characteristics of interventions [34,35]. Here, we outline the development of “C-Raven,” a single-session treatment engagement intervention for people with HIV who use drugs. The tablet-administered intervention is cross-platform compatible and uses a digital counselor to deliver information and teach behavioral skills based on cognitive behavioral therapy (CBT). The digital counselor interacts with the user in MI style through a series of menu-based conversations that allow for more personalized responses. Specifically, we describe the (1) conceptual model for behavior change, (2) translation to computer delivery including: user interface and digital counselor development, and (3) pilot testing methods [36-38].

## Methods

The intervention development was completed in 2 phases: background research and intervention development (Textbox 1).

**Development phases and pilot testing of the C-Raven Intervention for people with HIV and substance use.**

**Phase 1: Intervention background research**

- Model selection for theory-based intervention development
- Identify effective behavior change techniques from person delivered substance use interventions
- Identify behavior change techniques from technology-based interventions
- To increase relevance and engagement, identify key characteristics to culturally adapt intervention

**Phase 2: Intervention development**

- Intervention cognitive behavioral therapy (CBT) content and motivational interviewing (MI) “scripts” development
- Intervention scripts and MI style translation to computer delivery
- Software development, testing, and data storage
- Relevant, engaging digital counselor development and integration
- Mixed methods intervention testing of user experience

### Intervention Background–Phase 1

#### Model Selection for Theory-Based Intervention Development

We used Intervention Mapping to describe the stages of intervention planning [39]. For our theoretical framework and practical applications, we used the situated-information, motivation, and behavioral skills model because of its evidence base in HIV treatment adherence and substance use, its application to technology interventions, and its incorporation of sociodemographic factors that are essential to increasing engagement and adherence to substance use and HIV care [40-42].

#### Identify Behavior Change Techniques From Person Delivered Substance Use Interventions

Applying the IMB model, we developed a module that provided information on substance use and its effects on the body including HIV health outcomes [43]. We used behavior change therapies, primarily CBT and MI because of their strong evidence base in person-delivered substance use treatment and more recent adaptation to technology-delivered interventions [14,32,33]. MI was used to address the motivational component of IMB and we incorporated its nonjudgmental, collaborative counseling style which allows clients to explore and resolve ambivalence, explore a menu of change options, and identify their own reasons for behavior change [44]. From CBT, we incorporated exploration of self-talk about drug use and HIV, identification of high-risk moods and situations, and development of strategic coping behaviors to manage cravings and triggers for drug use [45].

#### Identify Behavior Change Techniques From Technology-Based Interventions

Applying the IMB model here, we reviewed technology-delivered interventions in HIV treatment which showed that CDIs need to increase information about HIV treatment adherence and promotion of patient-provider relationships [46]. From the literature on computer- and web-based interventions, we incorporated specific methods such as tailoring and normative feedback which build motivation for behavior change and therefore are potential mediators of improved outcomes [4,37,47-49]. Tailoring uses information from an individual to provide intervention material specific to that individual and normative feedback provides information to a subgroup using specific information about that subgroup [47-49]. Normative feedback about alcohol use and the quality of coping skills for substance use contributes to improved outcomes [48].

#### To Increase Relevance and Engagement, Identify Key Characteristics to Culturally Adapt Intervention

Our goal also was to develop a CDI that was relevant to and adapted for people with HIV who use drugs, primarily opioids. A culturally adapted intervention preserves the core components of an evidence-based treatment but translates key characteristics to be consistent with the ideas, values, beliefs, norms, attitudes, and knowledge of the target group [50]. The evidence suggests that adapting treatments can improve overall health outcomes [51,52]; however, there is relatively little information on cultural adaptations of technology-delivered interventions for substance use [53,54]. We therefore followed CDC [50] recommendations for cultural adaption using methods described previously to incorporate key characteristics including: specific HIV risk factors, neighborhood stressors, and alternatives to drug use [53,54].

### Intervention Development–Phase 2

#### Intervention CBT Content and MI “Scripts” Development

For phase 2, we conducted 34 in-depth interviews (IDIs) between July 2013 and May 2014 with purposively sampled people with HIV with active drug use and seeking care at an HIV clinic. The interviews were transcribed verbatim and coded independently by 2 investigators described elsewhere [55]. We explored patient experience with drug use (heroin or other opioids, cocaine, methamphetamine, or alcohol), substance use treatment history, knowledge gaps in HIV or hepatitis C virus, and drug use, and derived a lexicon of local phrases to be used by the digital counselor. Participants also provided information on: pros and cons of drug use, reasons to cut down or quit, positive coping strategies, strategies for improving medication and appointment adherence, and impact of drug use on provider-patient communication. These data were used to develop the content for the situated-information, motivation, and behavioral skills model and to develop counseling “scripts” that would be delivered by the digital counselor. The IDIs assessed technology use and access so that the intervention could be feasibly implemented with our patients. Most did not own a personal computer and therefore access would have to occur at the clinic. Finally, participants provided feedback on the appeal and acceptability of different images of potential digital counselors.

#### Intervention Script and MI Style Translation to Computer Delivery

Using information from the IDIs, CBT, MI, and clinical experience, we developed counseling scripts that were menu driven and allowed branching on client reported: gender, type of drug used (heroin, cocaine, methamphetamine, or alcohol), severity of drug use (based on the ASSIST) [56,57], types of triggers, consequences of drug use, mental health symptoms and client preference for: information topics, ways to manage triggers, problem-solving strategies, HIV treatment adherence strategies, and optional goal setting. The content of each intervention module incorporated our theoretical model as well as the specific evidence-based behavior change strategies. The use of treatment algorithms provided the opportunity to create an intervention that was highly tailored to client preference and to approximate MI style by providing choice. We also attempted to emulate MI approach by incorporating open-ended questions, affirmations, reflective listening, and summaries (Table 1). While we were not able to capitalize programmatically on the spontaneity of write-in answers for our CDI, carefully chosen content and lexicon were used to model a therapeutic interaction using a multiple-choice format. Each module contained a mixture of information and interaction with the program to recruit interest and engagement (further elaborated in the digital counselor section). To avoid a “lecture style,” information was conveyed using MI style of asking permission, using an informal tone, and obtaining feedback [58].

**Table 1:** Motivational interviewing style and adaptation to computer delivery.

MI^a^ technique	Translation
Nonjudgment	Digital counselor makes no positive or negative evaluations, only reflects user choices
Develop discrepancy	Elicits pros and cons of substance use
Ask permission	Digital counselor requests permission to proceed when information is to be presented
Express empathy	Digital counselor uses emotionally toned phrases, movements, and expressions; reflects participant answers to multiple choice questions
Emphasis on personal choice or readiness	Algorithmic branching depending on client choice
Strengthening commitment to change	Develop a change plan if desired; use of affirmations in response to multiple-choice answers
Evoke reasons to change	Digital counselor uses only open-ended questions, for example, asks what would change in client’s life if drug use were reduced or stopped

^a^MI: motivational interviewing.

Finally, we set the reading level at fifth grade and used large fonts for clarity to ensure that all users could navigate C-Raven. Intervention content and directions for navigating the intervention were read aloud by the digital counselor.

#### Software Development, Testing, and Data Storage

We identified technology partners in a local video game company and a user interaction designer and developer to create the intervention software and the digital counselor. We listed our system requirements (what we desired in the program) and that provided the basis for understanding what could be accomplished with the software partners. Frequent design reviews were used to measure progress against these requirements. Among our goals were to design a system that would allow for content flexibility through the use of independent content modules, ability to tailor intervention material according to the user’s ongoing responses, ability to insert pictures and videos, graphically designed to be highly interactive and engaging, and have the capacity to insert or build different digital counselors. We wanted a graphical user interface-based front end for editing scripts, such that changes could be made and immediately reviewed. Smaller but critical details were the ability for the digital counselor to refer to client answers given several modules back without having to create separate branches. Other features included: ability to pause or repeat a screen, buttons that illuminate when answered, database that collects not only responses but other variables such as time spent per screen. In addition, the CDI had to run on multiple platforms, for example, a tablet or desktop, and work on Mac, Android, or iOS. For data storage from the assessment and intervention, we used the university-based secure server. As we were asking about illegal drug use, the university server while expensive, provided the necessary level of security.

#### Relevant, Engaging Digital Counselor Development and Integration

We opted to include a digital counselor because this interface can increase interest, connection, and retention [59,60]. There is objective evidence that digital counselors must possess key characteristics to be effective [61-63]. Certain animations in facial features and gestures convey empathy, support, and acceptance while also directing attention to behavior change [64]. We worked with a graphic artist to produce sprite sheets (image files) where we could design and evaluate the addition of characteristics to make the digital counselors appear engaging and supportive. This included softened eyes by thickening the lid, open gestures, head nods, eyes that would half close, use of a warm gaze instead of a stare, movements that were of average speed, a slightly squat body, and a relaxed posture that conveyed an easiness to the appearance. In the IDIs, we piloted various digital counselors; participants selected a Raven digital counselor as the most appealing and they believed it would be appealing to other patients as well. A raven has significant local appeal because it is the mascot for the city’s professional football team. We also worked with the design artists to develop a familiar and acceptable background setting for the digital counselor. This included a desk in a hospital office as well as scenes from the city that would be familiar to all patients and contextualize the information being delivered. Participants preferred local city scenes and a purple background which is the iconic color of the football team. Selecting an acceptable computerized voice for the raven proved more difficult. High-quality voices were expensive; participants chose a voice that was a low alto or tenor but often found this voice to sound somewhat robotic. In the final phase, the team content experts mapped the digital counselor movements to the counseling script to ensure that MI characteristics were manifest

#### Mixed Methods Intervention Testing of User Experience

**Figure 1:**
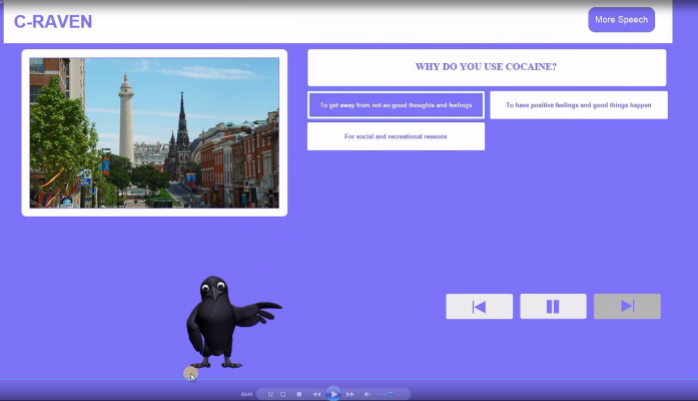
C-Raven study participant identifying triggers for using cocaine.

**Figure 2:**
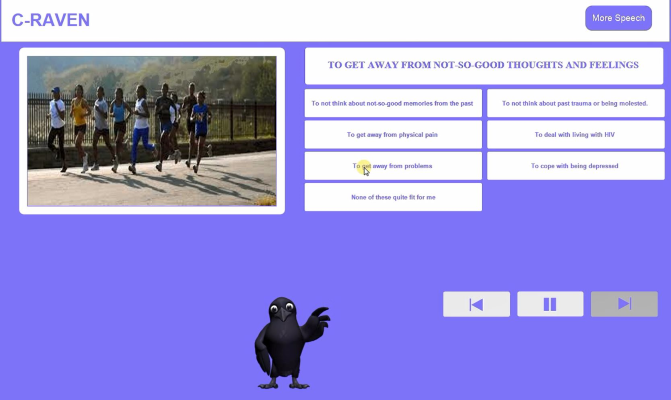
C-Raven study participant considers pros and cons of cocaine use.

Our final C-Raven to be tested was 1 session to be used as an engagement tool for people with HIV with substance use disorders. It used a Raven digital counselor that interacted with clients in MI style through a series of menu-driven conversations to present information, foster motivation and teach behavioral skills. The intervention is cross-platform compatible, usable on Windows, Mac, or iOS, and optimally viewed on a tablet or desktop. Screenshots of the digital counselor and a query menu are shown in Figures 1 and 2.

To evaluate user experience with the C-Raven intervention, we applied a mixed methods approach. We randomly selected 10 participants from the first round of IDIs to provide qualitative and quantitative feedback on C-Raven usability, content, and the Raven digital counselor.

To assess usability, we used the Think Aloud Protocol [65,66] and IDIs. The Think Aloud procedure examines program features while participants are concurrently completing the intervention. This protocol is widely used in product testing to gather spontaneous verbal and nonverbal behaviors of participants as they navigate a task. Responses are recorded verbatim, especially in the areas where any difficulty is encountered.

To assess program content and appearance, experience with the digital counselor, and to derive suggestions for improvement, we developed a semistructured interview guide to obtain responses to key questions on program acceptability, utility, and motivational impact. These interviews were completed in 45 minutes and were also audio recorded and transcribed verbatim. Any identifying information was removed from interview transcripts.

To quantitatively evaluate overall satisfaction with intervention components, we adapted an evaluation scale for CDI [67]. We added questions about the digital counselor (“Raven was patient”) based on recommendations for assessing embodied conversational agents [68] to develop a 29-item Likert-type “C-Raven Satisfaction Scale.”

#### Data Analysis

The IDI yielded responses to specific areas of the user experience: (1) usability, (2) content evaluation of the information presented and the utility of the behavior skills that were taught, and (3) digital counselor qualities. We developed an initial codebook using a deductive, a priori approach based on the dimensions of relevance to intervention evaluation. Using this initial codebook, HH and GC coded 3 transcripts independently and then met to refine the codebook and identify any emergent themes. They agreed on a final codebook, coded the remaining transcripts, and met to discuss and resolve discrepancies. Data from the Likert-type Satisfaction Scale was summed as a percentage of responses to each item.

### Ethical Considerations

All components of this study were reviewed for ethical compliance by Johns Hopkins University School of Medicine’s institutional review board (NA_00082481) and reviewed annually. Participants were identifiable only by study number. Participant data were only accessible by institutional review board–approved study team members who underwent yearly training and testing to ensure ethical conduct of research. Participants underwent informed consent that included a review of their understanding of the research procedures. They were informed that they could withdraw from the study at any time without impact on future care at the Johns Hopkins Medical Institutions and they could request that their data be removed from the analysis. The research team safeguarded the privacy of the participants to the extent permitted by the applicable laws and their duty to report.

The C-Raven intervention was compliant with the Health Insurance Portability and Accountability Act and the Personal Information Protection and Electronic Documents Act. The server was hosted in the Johns Hopkins cloud infrastructure to assure state and federal privacy and security regulations were met. Databases were stored on the Johns Hopkins OneDrive secure server. The intervention was delivered by a study tablet; no other IP addresses were used. All statistical analysis was conducted using anonymized data.

All study data will be submitted or published separately in JMIR Data [69].

## Results

### Overview

C-Raven was piloted among 10 individuals with HIV and active drug use (data on usability testing suggest that saturation in feedback occurs with 6-7 participants) [70]. Participants’ age ranged from 34 to 58 years, 60% (6/10) of them were female, 80% (8/10) of them identified as African American, and 70% (7/10) of them had high school or higher education. It was observed that 30% (3/10) of them used drugs daily, 20% (2/10) of them weekly or monthly, and the remaining used less than monthly. Computer use ranged from 50% (5/10) of participants reporting daily or weekly use to 30% (3/10) of them reporting no use.

### Usability Ratings

The program took an average of 17 (SD 3.16) minutes to complete. Results of the C-Raven Satisfaction Scale showed that the program was well received and all participants rated their program satisfaction at either a 4 (n=2) or a 5 (n=8) on a 5-point Likert scale. All participants endorsed the statement: “I would like to use the C-Raven program again.”

There were no reported difficulties navigating the system. One participant needed instruction using a mouse. Participants noted that among people attending the HIV clinic, internet use is low as is ownership of personal computers.

### Content Evaluation Results

The program evaluation IDI (Table 2) showed that participants found the C-Raven information useful, relevant, and nonjudgmental. Participants noted that the program presented information that they had not heard before, or heard in a new way. The information was considered relevant. The motivational quality of the intervention was also perceived positively because participants felt it allowed them to meaningfully reflect on the reasons for and the consequences of their drug use. The behavioral skills section was found to be useful by most participants; however, 1 participant wanted more emphasis on coping skills. The most consistent suggestion for improving the CDI was to provide even more information about the interaction of drugs with HIV and about the negative impacts of drug use. Participants opined that this would allow them to consider more broadly and more deeply the extent of the impact of drug use on their lives.

**Table 2:** C-Raven program evaluation based on in-depth interviews (n=10).

Construct	Sample quotations—positive	Sample quotations—negative or needs improvement
Program acceptability	104: “He does not lecture you. He informs you on ways where you get yourself together. He informs you if there is help. I felt that way cause the way it was talking to me, once I picked a topic, he went straight to it. It woke me up on that part.” 108: “I think it’s a healthy tool—I think a lot of the patients could use it—they’re not gonna just tell you stuff. Helps provider help patient.” 104: “...I definitely have a family member I’d like Raven to meet.”	104: “Raven could get down with more details on drug use. If we are talking about cocaine, the photos have to be about cocaine. The man lying on the street, cocaine does not do that to you. The Lexington market part that’s good. The man that dips down that was good.”
Program local relevance	101 I like how she (Raven) used words, like “mess with” 105: “I liked the shots of areas all throughout the program. It seemed like home. Some of these shots are fantastic.”	No improvements were provided to improve relevance
Utility of information	102: “I knew some things...hearing from computer was like hearing it for the first time.” 108: “How he gave you information about what cocaine does when you have HIV—how it affected your body.” 102: “It had all the reasons why I use drugs.”	101: “Add more info about HIV, add how to cope with HIV. More medical information about HIV, about how drugs can increase medical breakdown.” 102: ”At end of session, give patients resources” “Some of this information about drugs and HIV I have not heard before” 101: “—add how it suppresses the immune system—especially with crack and meth—more specifics on HIV”
Utility of behavioral skills	108: “Learned good ways of trying to not use the drug. Made me think about real reasons of why I did start using.” 103: “AVOID – COPE- ESCAPE—was the best thing. Add more time and have him talk a little more about that.”	109: “AVOID -COPE-ESCAPE is weak. I would like to see a “plug-in” so you can form your strategy to avoid using.“ 101: “The computer could probably be a little longer. The program could go longer. As far as more resolutions. How to problem solve a little bit more.”
Motivational impact	102: “...how many days not to get high made me think I could cut down 1 day. That maybe I can get it...it was touching...really touching to me” 108: “It gives you a little positive points in your life. Makes you reflect on some things. What the cocaine did to me stood out particularly.” 107: “He made me feel pushed to continue not to use drugs.” 102: “I can try harder not to use and I can do it.” 105: “It gets you to really thinking about the questions they’re asking—think about situations you put in and reasons why you are choosing to get high or choosing to use drugs.” 101: “I felt encouraged to quit, I did not feel obligated to...” 104: “How ACE (avoid, cope and escape skill) works that was more motivating than anything. Telling me what my trigger is, and how to spend my money, it said manicure lasts longer than a high.”	104: “ More on how, if we neglect ourselves, and keep using and keep going down the spiral staircase, how the results will really end up. It really needs to be slapped in the face with what the outcome is.” 102: “I could have been pushed more on the consequences.”

### Evaluation of Digital Counselor Quality and Qualities

We qualitatively and quantitatively assessed the acceptability and empathic quality of the digital counselor. Sample IDI quotations are shown in Table 3. The digital counselor was perceived to be nonjudgmental, understanding, motivating, and emotionally responsive. Most participants noted that they could be more open and truthful with a digital counselor. On the other hand, although participants perceived these qualities, a digital counselor was not necessarily seen as a substitute for a human counselor.

On the C-Raven Satisfaction Scale, 80% (4 or 5 on Likert scale) endorsed the statement “Raven understands what is hard about quitting drugs;” 70% of them endorsed the statement “Raven cares about me;” 1 participant endorsed the statement “Raven was harsh.”

**Table 3:** Participant feedback on the digital counselor.

	Feedback
Comparison to a person-delivered intervention	102: Question: “How does Raven compare with a counselor or provider?” Participant answer: “He is a provider!” 101: “It’s a good place for a person to start, but I would not want Raven to replace my therapist.” 103 “...Yeah because with computer it’s not as personal, there is no one to judge you.”
Ability to answer openly	107: “I would rather have the computer counseling...it gives you control. I felt like I could go in and be honest with the program.” 103: “Cartoon character makes it a lot easier because the cartoon does not express facial emotions that a doctor might when they are thinking about you. Cartoon makes you more truthful.”
Personal characteristics	107: “He was very sensitive and concerned. I picked up feelings of sensitivity and concern about the whole situation” 102: “I would not expect that he would respond, but he surprised me. The way he was answering the questions to me, I was like wow, he is all up in my head. He really answered the question for me.” 107: “Now, I think the size of the virtual counselor probably make a difference. He could be bigger.”

## Discussion

CDIs led by a digital counselor to reduce substance use among people with HIV may improve treatment access and improve health outcomes. We have described our development and pilot testing of C-Raven, a digital counselor–led, 1 session, brief (≈20 minute) treatment engagement intervention for people with HIV who use recreational drugs. The intervention is cross-platform compatible and usable on Windows, Mac, or iOS. We incorporated evidence-based practices and principles from both person- and technology-delivered behavior change interventions for substance use. Intervention “scripts” based on CBT and MI were developed, tested, and culturally adapted for people with HIV using qualitative and quantitative methods. Partnering with a user interaction designer and developer, we tested various digital counselors and program interfaces to create a locally appealing and relevant digital counselor, called C-Raven. C-Raven interacts with the client in MI style to present information, foster motivation and teach behavioral skills. The digital counselor-client conversation is menu driven and presents personalized content based on participants’ ongoing responses.

In pilot testing, C-Raven information and behavior skills were evaluated as useful and relevant. Because CDIs allow for the ability to process information anonymously through a new medium and at the participant’s own rate, they may facilitate knowledge acquisition [71]. Additionally, CDIs that are culturally adapted for the user, customized to the user’s reading, readiness, and computer skill levels, and that present information that is objective and relevant may improve knowledge acquisition as a component of behavior change [72].

Our study findings also indicate that participants viewed the Raven digital counselor as acceptable, empathic, and nonjudgmental. In person-delivered behavior change research, such qualities are considered essential to improving health outcomes [24]. Digital counselors can emulate these same characteristics if they are sufficiently anthropomorphic or empathic [60,73]. Importantly, participants also reported that because the digital counselor was nonjudgmental, they felt more comfortable, in control, and able to answer questions honestly than they would have to a human counselor. Although digital counselors and human counselors received little comparison in an RCT, our findings corroborate others showing that individuals can be more comfortable disclosing information and expressing sadness to a digital counselor [74]. This is a potentially critical element of digital versus person-delivered substance use treatment. It is well-established that substance use is the most highly stigmatized of all health issues [75,76]. Therefore, any method that potentially can break down barriers by reducing fear of stigma and facilitating therapeutic alliance could be instrumental in promoting behavior change.

The principal recommendations and limitations noted by participants were to increase the amount of information about the impact of drug use on HIV and more sessions to address drug use. We like others have found that systematic teaching about substance use using a computer exposes some of the gaps in patients’ knowledge about the harms of their use [77]. Furthermore, the complexity of treating substance use requires more therapeutic contacts as recent trials have questioned the efficacy of brief drug interventions [77]. We therefore plan to expand the number of sessions in the C-Raven package. Importantly, despite empathic, nonjudgmental qualities, the digital counselor was not necessarily viewed as a replacement for person-delivered counseling. Therefore, we also plan to include a community health worker module to (1) extend the C-Raven counseling and (2) facilitate connection to the person-delivered substance use treatment. Hybrid interventions potentially combine the strengths of both computer and person-delivered approaches in decreasing attrition and maintaining treatment effects with reduced provider time [78].

A notable limitation of our study is that our raven counselor was fashioned for a discrete community and therefore acceptability ratings may not generalize to other populations. Hence, we plan to test our “human” digital counselor using a different background setting against the Raven to assess the importance of patient choice and to compare acceptability and engagement with these 2 digital counselors across other populations of individuals with substance use. We also note that our digital agent interacts with the user using menu-based conversations. The recent work of Olafsson et al [79] to develop a natural language approach holds much promise for creating a more authentically empathic exchange between the digital counselor and the user. In addition, we used our own adaptations of measures to evaluate our pilot which may hinder generalizability. Future research will focus on systematically defining the key characteristics of digital counselors and the specific behavior change techniques that improve health outcomes. As part of this effort, the development of reliable and valid measures to assess CDIs, digital counselors, and human-computer interface experience is indicated.

This study developed and piloted an interactive, digital counselor–led CDI for substance use tailored to individuals with HIV. The process of developing a digital counselor–delivered CDI for behavior change necessarily requires integration from multiple disciplines including computer science, graphic design, psychology, and behavioral medicine research. Digital counselors appear acceptable and appropriate and may increase the effectiveness of interventions for substance use.

## Abbreviations

CBT: cognitive behavioral therapy
CDI: computer-delivered intervention
IDI: in-depth interview
MI: motivational interviewing
